# Catalytic preparation of levulinic acid from cellobiose via Brønsted-Lewis acidic ionic liquids functional catalysts

**DOI:** 10.1038/s41598-018-38051-y

**Published:** 2019-02-12

**Authors:** Shiwei Liu, Kai Wang, Hailong Yu, Binghan Li, Shitao Yu

**Affiliations:** 1 College of Chemical Engineering, Qingdao University of Science and Technology, Qingdao, 266042 China; 2 Institute of Chemical Industry of Forest Products, CAF, Nanjing, 210042 China

## Abstract

Brønsted-Lewis acidic ionic liquids (ILs) were applied to catalyze cellobiose to prepare levulinic acid (LA) in one pot under hydrothermal conditions. Under the optimum conditions, the highest LA yield of 67.51% was obtained when low [HO_3_S-(CH_2_)_3_-mim]Cl-FeCl_3_ (molar fraction of FeCl_3_ x = 0.60) was used. This indicated the Brønsted-Lewis acidic ILs played an active role in the conversion of cellobiose to LA. The catalytic mechanism of ILs had been established, disclosing that the Brønsted-Lewis acidic ILs had the catalytic synergistic effect originating from its double acid sites. During the reaction process, the Lewis acid sites improved the isomerization of glucose to fructose, then the Brønsted and Lewis acid sites simultaneously enhanced the dehydration of fructose to produce hydroxymethylfurfural (HMF), which was propitious to the synthesize LA with high yield. In addition, LA could be easily extracted by methyl isobutyl ketone (MIBK), and the ILs could retain its basic activity after 5 cycles. The solid residues were characterized using SEM, FT-IR and TG-DTG spectroscopy. It was the conclusion that a large amount of humins were produced during the cellobiose conversion process. In this reaction, the ILs not only overcomes the problems of the conventional catalyst, but also completes the reaction-separation integration and the recycling of the catalyst. This paper provided an important theoretical basis for the application of ILs in the field of biomass.

## Introduction

As the main driving force for human survival, energy is very important for the development of society^1^. But with the increasingly diminishing of non-renewable resources such as coal and oil, the modern society is facing a serious crisis of resources and energy^2^. Biomass as a kind of renewable resources has the advantage of low-cost, various kinds and conducive to the realization of carbon cycle^3^. Recently, exploring how to produce energy and chemicals from biomass resources in order to meet the human energy needs of the society development is regarded as the important goal^4,5^. As one of the 12 platform chemicals, levulinic acid (LA) could be produced from biomass resource^6–11^. LA is an active compound with both ketone and carboxylic structure and can undergo chemical reactions such as esterification, redox, substitution and polymerization^12–14^. Due to this specific nature, LA has been widely used in pharmaceutical products, pesticides, lubricant additives, resins, coatings, dyes, solvents, rubber and plastic additives and surfactants and other industries^15^. Therefore, studying how to produce LA from biomass has become one of the hot areas for biomass conversion.

Cellulose is the most abundant biomass resource, but it is not easily degraded due to its special hydrogen bond structure and poor water solubility^16^. Therefore, production of LA from cellobiose has become one of the important steps in biomass conversion. Cellobiose is a product of cellulose hydrolysis which is composed of glucose units linked together by β-glycoside bonds. The traditional method for the conversion of biomass to LA was inorganic acid catalysis. Such as, HCl produced 50 mol% LA by catalyzing glucose at 180 °C^17^. But the problems were that corrosion of equipment, complicated separation procedure, environmental problem, and not recyclable of catalyst^18^. To overcome these disadvantages, a number of solid acid catalysts were used to the process of biomass conversion to LA. Zuo *et al*. found out that CP-SO_3_H showed good catalytic when it had been used to produce LA from cellobiose. In 10 h at 170 °C cellobiose could be effectively converted to LA with a yield of 38.2% and glucose of 21.2% yield was achieved. It was suggested that its ability to adsorbs/attract cellobiose and to disrupt hydrogen bonds of cellobiose^19^. Yang *et al*. proposed a solid acid catalyst SO_4_^2−^/SnO_2_ to convert cotton cellulose, leading to 11.0% *wt* yield of hydroxymethylfurfural (HMF) and 26.8% *wt* yield of glucose, respectively^20^. Gomes *et al*. reported that H_3_PW_12_O_40_ was used as catalyst and Dimethyl sulfoxide (DMSO) was used as solvent that enabled catalytic conversion of fructose to HMF which 92% yield was obtained at 120 °C^21^. However, the recycling of the catalyst and restricting accessibility to the matrix-bound catalytic sites were shortcoming. Therefore, the study of easy-to-recover and environmentally-friendly catalysts is crucial to improve the productivity of LA nowadays.

As a kind of novel reaction medium, Ionic liquids (ILs), were seen as environmental-friendly catalyst. Simultaneously, ILs also had the advantages of adjustable acidity, easy recovery, and stable structure^22^. Swatloski *et al*. firstly discovered that cellulose could be dissolved up to 25% in the presence of [C_4_mim]Cl, which opened up the possibility of ILs were used in the field of biomass conversion^23^. Bao *et al*. used ILIS-SO_3_H and ILIS-SO_2_Cl as a catalyst to prepare HMF from fructose, leading to a yield of 70.1% and 67.2% for HMF, respectively^24^. Li and Zhao hydrolyzed cellulose leading to 43% yield of glucose and 77% yield of TRS, using H_2_SO_4_ in [Emim]Cl under at 100 °C reactions 9 h^25^. Ding’s group reported that [C_4_SO_3_Hmim][CH_3_SO_3_] was used as a catalyst and CuCl_2_ was used as a co-catalyst to catalyze the conversion of microcrystalline cellulose to HMF, leading to the yield of 87.6% in a single step^26^. Tao *et al*. used [SO_3_Hmim][HSO_3_] as catalyst and MnCl_2_ as co-catalyst to prepare LA from fructose, leading to HMF and LA of 47.5%, 10.5%, respectively^27^. Shen *et al*. reported that the conversion of cellulose to LA using [BSmim]HSO_4_ as catalyst, which was obtained by 25.5% yield under 2 h without forming too much by-products^28^. These results suggested that synergistic catalysis of ILs and metal chloride played a positive role in the development of an effective process for biomass conversion. Unfortunately, so far, Brønsted-Lewis acidic ILs had a litter reports in the field of biomass conversion. In our laboratory, some Brønsted-Lewis acidic ILs were synthesized which were used as a catalyst for the production of HMF from fructose that 93.4% yield of HMF was obtained, simultaneously, the ILs recovered five times still maintained this good catalytic activity^29,30^. It was indicated that Brønsted-Lewis acidic ILs had good catalytic activity and stability, and Brønsted-Lewis acidic ILs were easier to recycle than mixed acid systems.

In this study, Brønsted-Lewis acidic ILs were employed in the catalytic conversion of cellobiose to LA. Moreover, the effect of various reaction conditions such as time, temperature and dosage of ILs was also investigated on the cellobiose conversion to LA. The recovery performance of ILs and separation of LA was also investigated in order to optimize the cost of the process. Here, we proposed the catalytic mechanism of ILs, revealing the catalytic effect of Brønsted and Lewis acid sites. It was intended to provide an important theory for the development of highly active and highly selective catalysts in the process of biomass resource catalytic conversion.

## Experimental

### Materials

Cellobiose (99%, Shanghai, China), Glucose (97%, Shanghai, China), LA (99%, Tianjin, China), HMF (96%, American), Methyl isobutyl ketone (MIBK) (98%, Tianjin, China), 1,3-propane sultone (99%, Beijing, China), H_3_PW_12_O_40_ (99%, Beijing, China).

### Catalytic study

**Figure 1:**
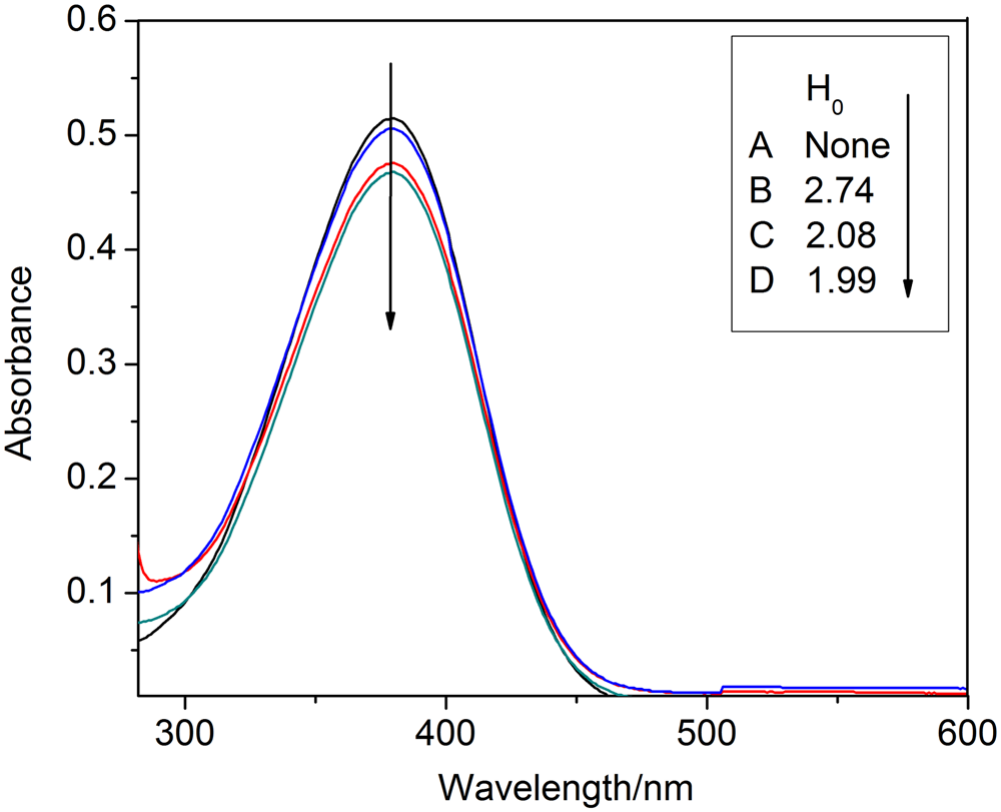
UV-visible spectroscopy of p-nitroaniline in water. (A) Blank (B) [HO_3_S-(CH_2_)_3_-NEt_3_]Cl, (C) [HO_3_S-(CH_2_)_3_-py]Cl, (D) [HO_3_S-(CH_2_)_3_-mim]Cl.

Brønsted-Lewis acidic ILs were prepared using previously reported method^30^. The synthetic reaction path of [HO_3_S-(CH_2_)_3_-NEt_3_]Cl-ZnCl_2_ (see Supplementary Fig. S1). The 3-(trieth-ylamine-N-yl)-propane-1-sultonate ([SO_3_-(CH_2_)_3_-NEt_3_]) was prepared by reaction triethylamine (0.5 mol) and 1,3-propane sultone (0.5 mol) at 75 °C for 3 h, and then [SO_3_-(CH_2_)_3_-NEt_3_] (0.1 mol) and HCl (0.1 mol) were reacted for 2 h at 90 °C, (3-sulfonic acid)-Propyltriethylammonium-um chloride ([HSO_3_-(CH_2_)_3_-NEt_3_]Cl) was obtained. [HSO_3_-(CH_2_)_3_-NEt_3_]Cl-ZnCl_2_ was obtained by the reaction of [HSO_3_-(CH_2_)_3_-NEt_3_]Cl with different mole numbers of ZnCl_2_ for 3 h at 80 °C. Imidazole and pyridine ILs were prepared according to the above method. Three kinds of Brønsted acidic ILs were characterized by UV-visible spectroscopy (Fig. 1), pH (Table 1), IR (see Supplementary the IR spectral data of ILs), ^1^H NMR (see Supplementary Fig. S2), respectively.

**Table 1:** The yield of LA using different catalysts.

Entry	IL	Yield of the main products (%)			pH
		Glucose	HMF	LA	
1	[HO_3_S-(CH_2_)_3_-mim]Cl	5.17	0	50.19	2.95
2	[HO_3_S-(CH_2_)_3_-mim]Cl-ZnCl_2_ (x = 0.60)	8.20	0	65.04	3.17
3	[HO_3_S-(CH_2_)_3_-mim]Cl-FeCl_3_ (x = 0.60)	10.85	0	67.51	2.37
4	[HO_3_S-(CH_2_)_3_-mim]Cl-CrCl_3_ (x = 0.60)	2.10	1.03	27.21	3.64
5	[HO_3_S-(CH_2_)_3_-NEt_3_]Cl	4.27	0	43.19	3.58
6	[HO_3_S-(CH_2_)_3_-NEt_3_]Cl-ZnCl_2_ (x = 0.60)	6.10	0	63.16	3.56
7	[HO_3_S-(CH_2_)_3_-NEt_3_]Cl-FeCl_3_ (x = 0.60)	7.50	0	65.23	2.54
8	[HO_3_S-(CH_2_)_3_-NEt_3_]Cl-CrCl_3_ (x = 0.60)	0	0.84	36.40	4.00
9	[HO_3_S-(CH_2_)_3_-py]Cl	4.53	0	43.19	3.34
10	[HO_3_S-(CH_2_)_3_-py]Cl-ZnCl_2_ (x = 0.60)	6.67	0	65.16	3.53
11	[HO_3_S-(CH_2_)_3_-py]Cl-FeCl_3_ (x = 0.60)	9.39	0	67.23	2.49
12	[HO_3_S-(CH_2_)_3_-py]Cl-CrCl_3_ (x = 0.60)	2.10	0.97	36.40	3.69
13	Blank	1.95	0.32	3.17	—
14	FeCl_3_	1.70	20.51	17.11	—
15	HCl	5.82	0	55.77	—
16	H_3_PW_12_O_40_	6.11	0	60.94	—

### Catalytic properties tests

In the typical procedure, cellobiose (0.50 g), ILs (0.62 mmol) and de-ionized water (30 ml) were mixed in the stainless steel autoclave and reacted at 180 °C for 10 h while maintaining vigorous stirring during the reaction. After the reaction, stainless steel autoclave was rapidly cooled in water. The solid residues were separated from the liquid products by filtration. LA product and IL could be easily separated by extraction with MIBK. Cellobiose and solid residues were characterized using DTG-TG, FT-IR, SEM. LA and HMF were analyzed by HPLC, which was equipped with UV detection and an ODS-EP C_18_ reversed-phase column (4.6 × 250 mm, Intersil). Detection conditions: mobile phase 15 mmol/L acetonitrile and phosphoric acid-sodium dihydrogen phosphate buffer solution (v_1_:v_2_ = 15:85), flow rate 1.0 ml/min, column temperature 30 °C. Glucose was analyzed by DNS method. The LA, HMF and glucose yield was calculated as equation (1):

1 $${\rm{Yield}}( \% )=\frac{\mathrm{The}\,\mathrm{number}\,\mathrm{of}\,\mathrm{moles}\,\mathrm{of}\,\mathrm{product}({\rm{mol}})}{\mathrm{The}\,\mathrm{number}\,\mathrm{of}\,\mathrm{glucose}\,\mathrm{in}\,\mathrm{the}\,\mathrm{cellobiose}({\rm{mol}})}\times 100 \% $$

### Characterization

Acidic ILs were characterized using FT-IR, UV-visible, ^1^H NMR, pH. FT-IR spectra were recorded in the wavelength range of 4500–400 cm^−1^ on a Nicolet 510 P FT-IR spectrometer using the KBr method. UV-visible spectroscopy was recorded in the wavelength range of 600–200 cm^−1^ on PGENERAL TU-1810. P-nitroaniline was used as indicator, and water was used as solvent (at a 5 mg/ml concentration in water), and ILs concentration (at a 25 mmol/L in P-nitroaniline water). Brønsted-Lewis acidic ILs were determined by pH value using pHs-25, and ILs concentration (at a 20 mmol/L in water). The ^1^H NMR spectra were recorded on a Bruker AV500 Fourier-transform with reference to SiMe_4_ using solvent D_2_O. Cellobiose and solid residues were characterized using DTG-TG, FT-IR and SEM. DTG-TG was done using a Mettler Toledo model SDTA 815 under nitrogen flows from 25 °C to 600 °C with a heating rate of 10 °C min^−1^. SEM images were recorded on Hitanchi 5–4800 field emissions scanning electron microscopy.

## Results and Discussion

### The acidity of ILs

The Brønsted acidity of three kinds ILs was determined by UV-visible spectroscopy. The acidity of ILs was calculated by the Hammett equation H_0_ = pK(I)_aq_ + log([I]/[IH]^+^), which indirectly reflects the relationship of acidity and activity. As shown in Fig. 1, for ILs with the same chloride anion structure, the acidity of the ILs increased in order: d(imidazolyl) > c(pyridyl) > b(triethylamine).

### Effects of catalysts on the LA yield

ILs were considered as desirable catalyst and their structures were changed by adjusting the cation and anion in order to obtain the desired properties. According to Ren’s report^31^, anion played a key role in the process of biomass conversion to LA, such as HSO_4_, H_2_PO_4_, CH_3_SO_3_, Cl^−^. Zuo *et al*.^19^ found that Cl^−^ could better combine with the hydroxyl of cellulose, increasing the breakage of the hydrogen bonding network of cellulose to break the extensive and facilitate its dissolution. However, the CP-SO_3_H catalyst used by Zuo *et al*. was easily deactivated under hydrothermal conditions. Therefore, in this study, different kinds of Brønsted-Lewis acidic ILs were used to catalyze the conversion of cellobiose to LA. As shown in the Table 1, [HO_3_S-(CH_2_)_3_-mim]Cl, [HO_3_S-(CH_2_)_3_-NEt_3_]Cl, [HO_3_S-(CH_2_)_3_-py]Cl combined three kinds of metal salts, ZnCl_2_, FeCl_3_, and CrCl_3_ were used in this study. When the ILs had the same cation structure, their acidity was affected by the metal chloride, the acidity of ILs increased in order: CrCl_3_ < ZnCl_2_ < FeCl_3_. The acidity of the Brønsted acidic ILs increased in order: [HO_3_S-(CH_2_)_3_-NEt_3_]Cl < [HO_3_S-(CH_2_)_3_-py]Cl < [HO_3_S-(CH_2_)_3_-mim]Cl. Simultaneously, compared with [HO_3_S-(CH_2_)_3_-py]Cl and [HO_3_S-(CH_2_)_3_-NEt_3_]Cl, [HO_3_S-(CH_2_)_3_-mim]Cl had good catalytic performance. When [HO_3_S-(CH_2_)_3_-mim]Cl-FeCl_3_ (x = 0.60) was tested, the LA yield was 67.51% at 180 °C for 10 h. In the presence of Lewis acidic CrCl_3_, Brønsted-Lewis acidic ILs had poor catalytic effect. This was because CrCl_3_ could hinder the formation of LA^32^. Consequently, the stronger catalytic activity of these ILs, the higher yield of LA was obtained, which indicated that the acidity of ILs played key role in the process of catalyzing the conversion cellobiose to LA. This phenomenon was caused by the reason that the stronger acidic. The more would promote the protonation of glycoside oxygen. Simultaneously, HCl and H_3_PW_12_O_40_ were also used as reference acid catalysts for conversion of cellobiose to LA, and gave 55.77% and 60.94% yields of LA, respectively. Compared to [HO_3_S-(CH_2_)_3_-mim]Cl-FeCl_3_ (x = 0.60), HCl and H_3_PW_12_O_40_ had poor catalytic effect.

**Figure 2:**
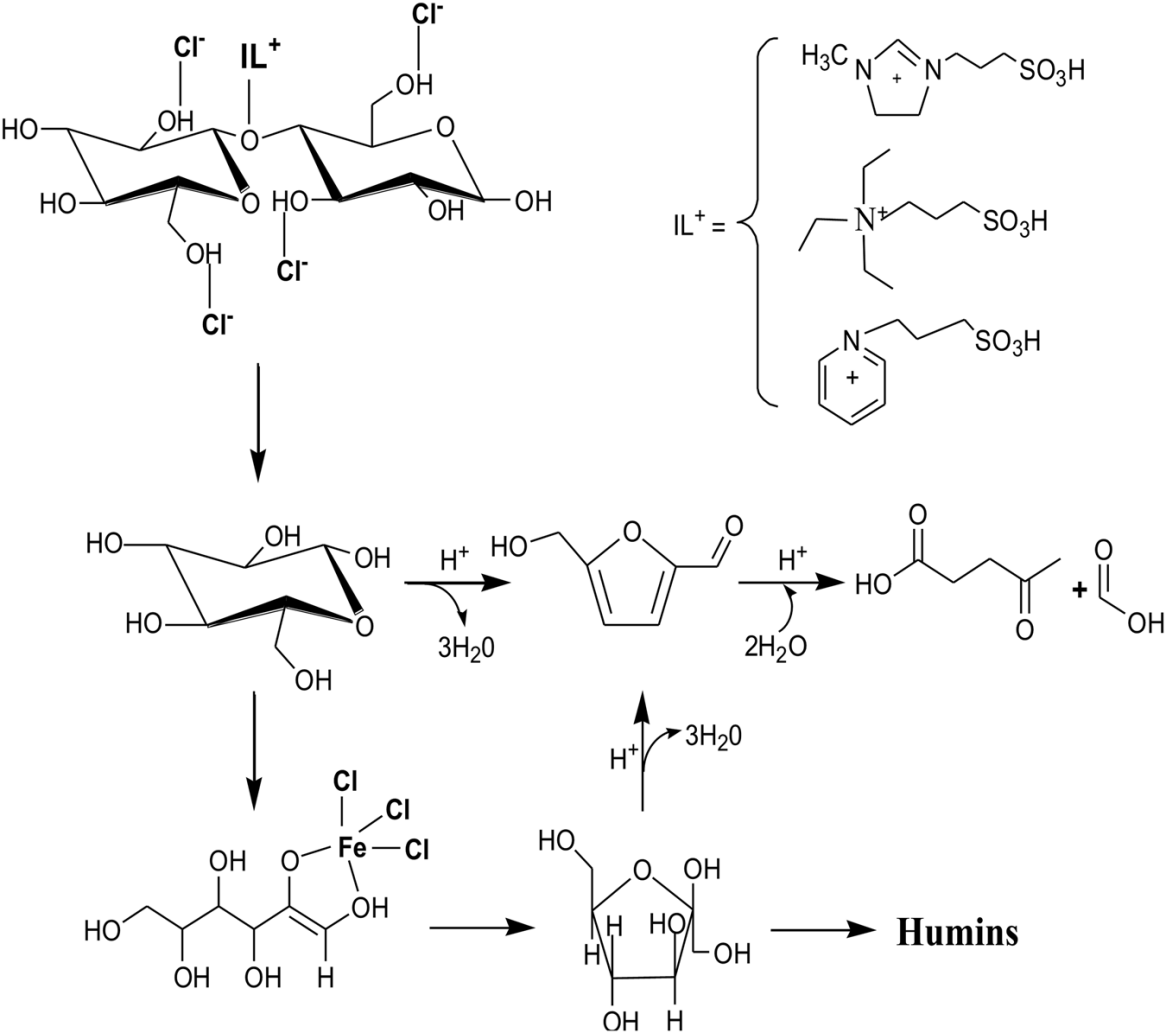
Mechanism of cellobiose conversion to LA.

A series of complicated reactions were included in the conversion of cellobiose to LA, which involved depolymerization, dehydration, isomerized and so on. The conversion of cellobiose to LA was divided into three intermediate reaction steps. As shown in Fig. 2, the cellobiose was depolymerized into glucose at first and then glucose was converted to HMF, which further was hydrolyzed to LA. In the first step, Cl^−^ was attached to the hydroxyl groups of cellobiose by hydrogen-bond interaction, and then cellobiose was adsorbed onto the catalyst. Thereby β-glycoside bonds were broken down by protonation. In the second step, there were two ways to produce HMF from glucose, one was that HMF was directly produced from glucose, another was that glucose isomerized to fructose and then to produce HMF from fructose. According to the literature^33^, glucose isomerized to fructose was the control step of the whole reaction, and Lewis acidic could promote this process. In the third step, HMF was directly hydrolyzed to produce LA under the action of H^+^. Table 1 and Fig. 2 shown that apart from acidity factor, the synergistic catalytic of Brønsted-Lewis not only could break the glycoside bond of cellobiose, but also could provide enough acid sites to promote the conversion of glucose to LA. At the same time, since H_3_PW_12_O_40_ included metal W and P elements which could provide Lewis acidic, and H^+^ which could provide Brønsted acidic, so LA also had good yield. However, due to H_3_PW_12_O_40_ lack Cl^−^, the first step in the conversion of cellobiose to LA could be affected, so its catalytic effect was lower than that of most Brønsted-Lewis acidic ILs.

### Effects of reaction conditions on the LA yield

Table 2 shows the effect of different reaction conditions on the conversion of cellobiose to LA. The reaction temperature was very important for preparing LA from cellobiose. With increasing of reaction temperature from 160 °C to 180 °C (Entry 1, 2, 3), the yield of LA increased from 51.0% to 67.51%, the yield of glucose and HMF decreased from 12.74% to 10.85% and from 1.47% to 0%, respectively. However, when the temperature exceeded 180 °C (Entry 4), the yield of LA declined to 59.72%. This was because that as the temperature increased, the viscosity of the reaction system would fall, the mass transfer rate would increase, and the reaction rate also would increase, which was beneficial to the generation of LA^28^. However, glucose and HMF were easily polymerized into by-products when a high temperature was employed^34^. Therefore the yield of LA decreased as the temperature was more than 180 °C.

**Table 2:** The yield of LA using different reaction conditions.

Entry	*T*/°C	*t*/h	IL/mmol	Yield of the main products (%)		
				Glucose	LA	HMF
1	160	10	0.62	12.74	51.0	1.47
2	170	10	0.62	10.92	58.50	0.62
3	180	10	0.62	10.85	67.51	0
4	190	10	0.62	8.75	59.72	0
5	180	6	0.62	13.78	50.88	3.35
6	180	8	0.62	11.39	56.43	1.71
7	180	12	0.62	10.14	58.73	0
8	180	10	0.32	6.37	45.01	0.83
9	180	10	0.86	11.57	64.97	0
10	180	10	1.24	12.00	63.50	0

Reaction time also was an important factor which affected conversion of cellobiose. With the extension of reaction time (Entry 5, 6), the yield of the LA arranged from 50.88% to 56.43%, but the yield of glucose and HMF decreased correspondingly. When reaction time reached 10 h, the maximum LA yield of 67.51% was achieved (Entry 3). However, when the time exceeded 10 h (Entry 7), the yield of LA declined to 58.73%, the yield of glucose declined to 10.14% and the yield of HMF was still 0%. This might because the cellobiose could not be fully reacted in a short reaction time, and LA was degraded into other products when reaction time was too long.

Table 2 also shows the effect of the catalyst dosage on LA yield. When the dosage of ILs increased from 0.32 mmol to 0.62 mmol (Entry 8, 3), the yield of LA increased from 45.01% to 67.51% obvious. This increased could be attributed to an increase in the availability of the number of active sites. Notably, the yield of LA decreased from 67.51% to 63.50% when more than 0.62 mmol of ILs were added (Entry 9, 10), which suggested that excessive amount of catalyst would accelerate the decomposition rate of LA, thus leading to the decrease of LA yield. It was implied that sufficient catalytic sites were available for conversion of cellobiose to LA at the experimental conditions. Meanwhile, with the increase of catalytic sites, the degradation rate of cellobiose increased, so the glucose yield increased slightly.

### Recycling of IL

**Figure 3:**
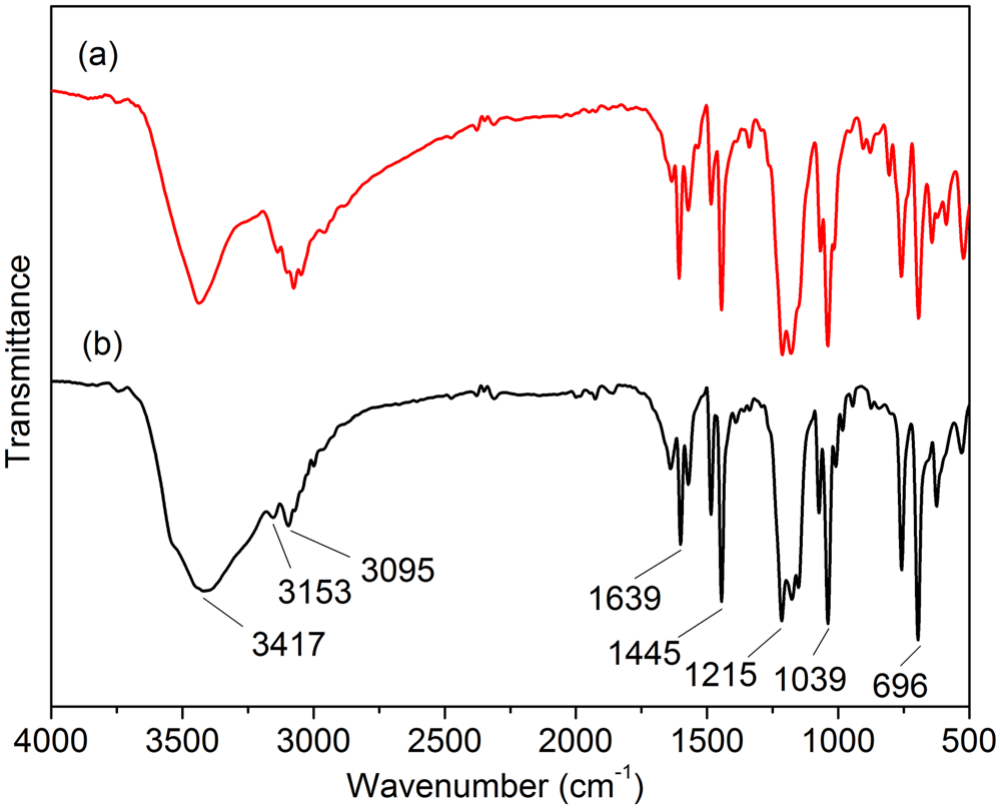
IR spectrum of IL. (**a**) IL recycled after five times and (**b**) fresh IL, [HSO_3_-(CH_2_)_3_-mim]Cl-FeCl_3_ (x = 0.60).

**Figure 4:**
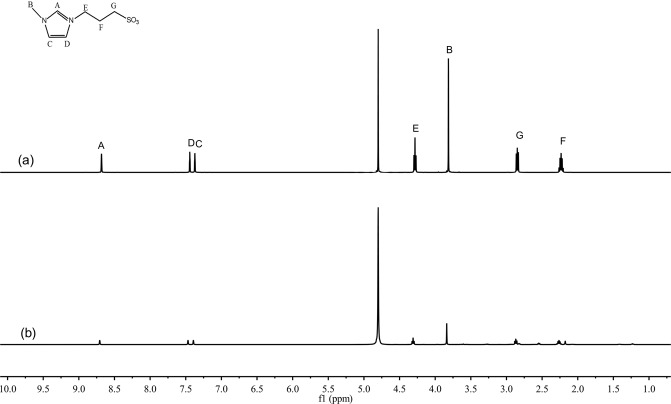
Typical ^1^H NMR spectra. (**a**) fresh IL and (**b**) IL recycled after five times, [HSO_3_-(CH_2_)_3_-mim]Cl-FeCl_3_ (x = 0.60).

ILs were an environmentally-friendly catalyst due to their respectability and reusability. The IL recycled after five times and fresh IL was investigated by IR spectra (Fig. 3), the bands of 1445 cm^−1^ and 1039 cm^−1^ represented asymmetric stretching vibration and symmetric stretching vibration of sulfonic acid, respectively. Bands of 3417 cm^−1^, 3153 cm^−1^ and 3095 cm^−1^ represented hydrogen bond of sulfonic acid. We could see that the band around 3340 cm^−1^ had obviously changes, corresponding to H^+^ of sulfonic acid was lost in the catalytic process. Through the ^1^H NMR spectra of fresh IL and IL recycled after five times was detected, as shown in Fig. 4. From Fig. 4, it could be seen that the IL catalyst remained stable under the reaction conditions and did not decompose to the corresponding zwitterion. As shown in Table 3, the yield of LA slightly declined from 67.51% to 62.57% after [HO_3_S-(CH_2_)_3_-mim]Cl-FeCl_3_ (x = 0.60) was used repeatedly five times. Thus, Brønsted-Lewis acidic ILs were a stable, highly reproducible catalyst.

**Table 3:** Effect of different reusability of catalyst on LA yield.

Cycle	Yield of the main products (%)		
	Glucose	LA	HMF
1	10.85	67.51	0
2	9.95	63.93	0
3	10.40	64.72	0
4	10.11	62.91	0
5	9.91	62.57	0

### Separating of LA

**Figure 5:**
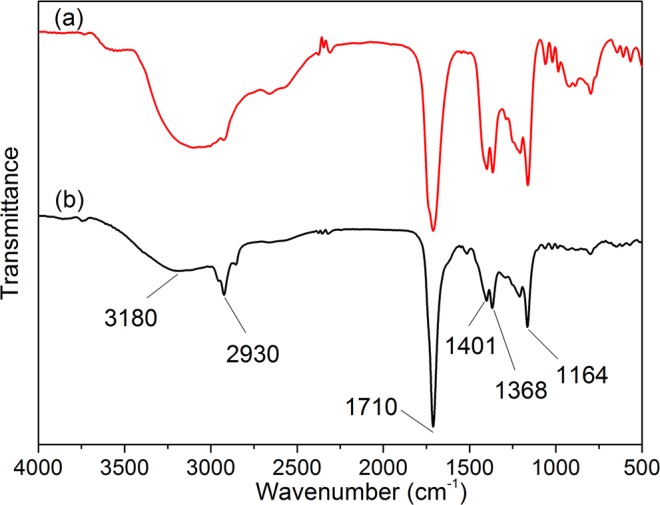
IR spectrum of LA. (**a**) LA standard and (**b**) LA product.

**Figure 6:**
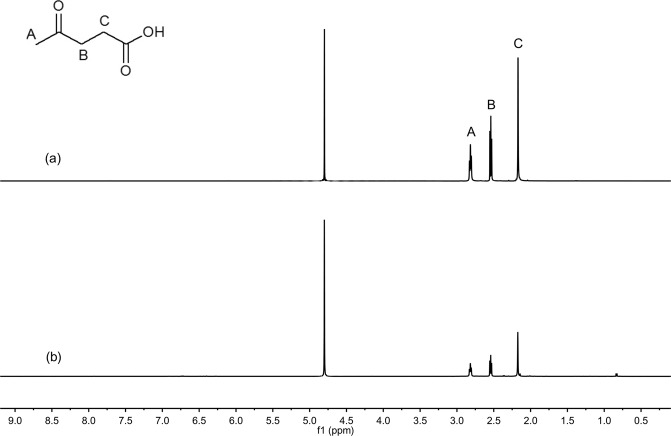
Typical ^1^H NMR spectra. (**a**) LA standard and (**b**) LA product.

LA product could be easily separated by extraction with MIBK. Through comparison ^1^HNMR and IR of LA standard and LA product were detected, as shown in Figs 5 and 6. From Fig. 5, two bands at 3180 cm^−1^ and 2930 cm^−1^ changed noticeably, corresponding to O-H and C-H stretching vibrations, respectively. This might be caused by the reaction of aldehydes and formic acid. In comparison, the band at 1710 cm^−1^ didn’t change very much, which was attributed to C=O stretching vibration. From Fig. 6, we could see that the characteristic peaks of the recovered LA and LA product were similar. So Figs 5 and 6 confirmed that LA could be separated in this reaction system.

### Characterization of solid residues

In the process of LA production, and a lot of humins would be produced and the humins could be characterized by IR spectra (see Supplementary Fig. S3). The absorption peak at 3400 cm^−1^ was attributed to O-H stretching vibrations. 2924 cm^−1^, 1694 cm^−1^ and 1621 cm^−1^ corresponding to C-H, C=O, C=C stretching vibrations, respectively. The absorption peaks at the 1000–1500 cm^−1^, which were attributed to C-O stretching and O-H bending vibrations. The absorption peaks at 745–880 cm^−1^, which were attributed to aromatic C-H out-of-plane bending vibrations. These were similar to the literature record^29^.

**Figure 7:**
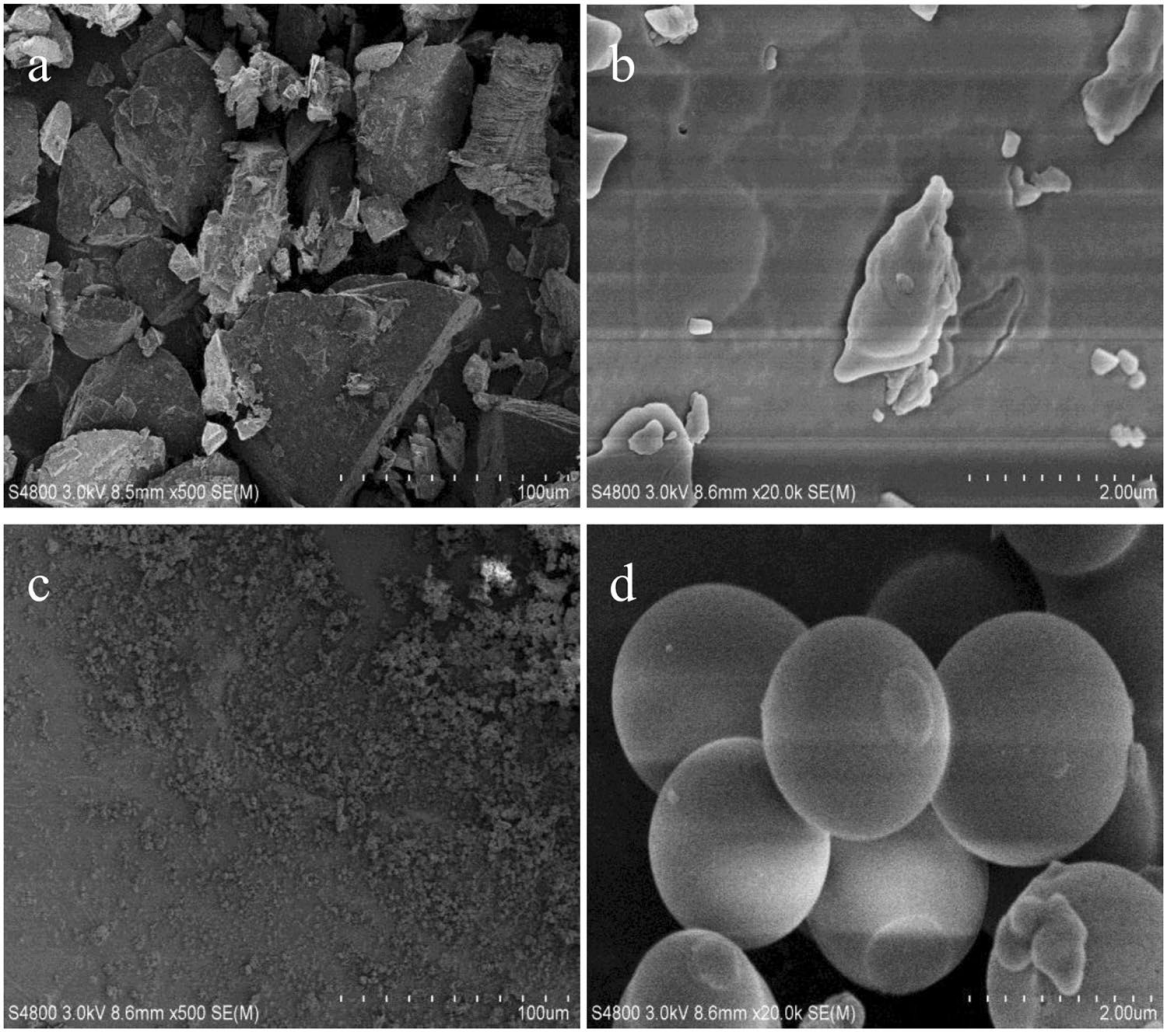
SEM images of cellobiose and solid residues. (**a**,**b**) cellobiose and (**c**,**d**) solid residues.

To further reveal the difference between solid residues and cellobiose, we had performed SEM characterization. Figure 7a indicated that the particle size of cellobiose lower magnification with a distinctly massive structure, and cellobiose surface also had a sporadic block structure under higher magnification as shown in Fig. 7b. After the reaction, the original block structure was decomposed leaving a small agglomerate particle as shown in Fig. 7c,d at higher magnification exhibited independent spherical particle. According to Ren’s report^31^, characteristic morphology of solid humins substances was spherical particle at higher magnification. It was indicated that the solid residues were humins under the reaction system.

**Figure 8:**
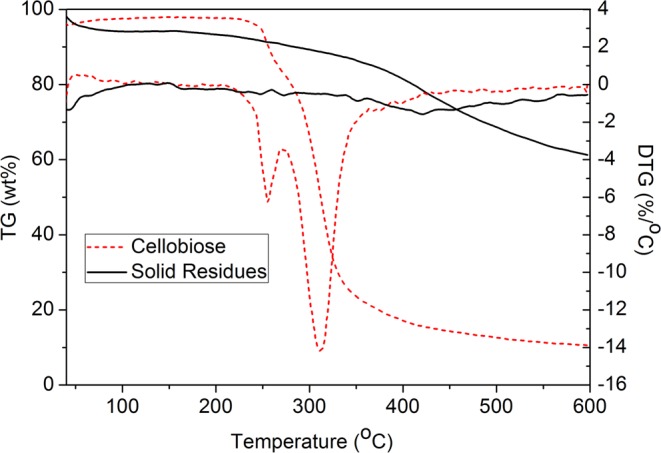
TG-DTG curves of cellobiose and solid residues.

The changed in the weight of the cellobiose and solid residues as a function of temperature was shown in Fig. 8. It was clearly to see that the main decomposition temperature of cellobiose in the range of 245–334 °C. After reaction, the cellobiose peak disappeared in the range of 245–334 °C. Simultaneously, a broader peak appeared between 341 °C and 499 °C. According to literature reports^35^, the appeared of characteristic peak in the range of 341–499 °C was solid humins. It was indicated that the thermal stability of solid humins substances was excellent, mainly because of increased heat resistance of humins after further dehydration in the conversion process.

## Conclusions

The conversion of cellobiose to LA was investigated using Brønsted-Lewis acidic ILs as catalyst. [HO_3_S-(CH_2_)_3_-mim]Cl-FeCl_3_ (x = 0.60) was of good catalytic property and the yield of LA was 67.51%. The synergistic effect originating from the Brønsted and Lewis acid sites of IL enhanced the IL catalytic performance significantly. The results strongly suggested that Brønsted-Lewis acidic ILs could play an important role in exploring new efficient and easy to use processes for the production of LA. LA could be extracted by MIBK, and IL could retain its basic activity after 5 cycles. Through SEM, DTG-TG, and IR investigations on the solid residues, it was conclusive that a large amount of humins were produced during the cellobiose conversion process. This study was highly useful for the development of a clean and environmentally-friendly for cellobiose conversion into high value platform chemicals LA.

## Supplementary information

Catalytic preparation of levulinic acid from cellobiose via Brønsted-Lewis acidic ionic liquids functional catalysts

## Data Availability

All data generated or analysed during this study are included in this published article (and its Supplementary Information files).
