# Supplementary material for: Catalytic preparation of levulinic acid from cellobiose via Brønsted-Lewis acidic ionic liquids functional catalysts
Source: Sci Rep. 2019 Feb 12;9:1810. doi: 10.1038/s41598-018-38051-y (PMC6372595; doi:10.1038/s41598-018-38051-y)
Supplement: Supplementary file 1 — Catalytic preparation of levulinic acid from cellobiose via Brønsted-Lewis acidic ionic liquids functional catalysts [file 41598_2018_38051_MOESM1_ESM.docx]

**Catalytic preparation of levulinic acid from cellobiose via Brønsted-Lewis acidic ionic liquids functional catalysts**

**Shiwei Liu^a,b,*^^[[1]](#footnote-1)^, Kai Wang^a^, Hailong Yu^a^, Binghan Li^a^, Shitao Yu^a*^^[[2]](#footnote-2)^.**

^a^ College of Chemical Engineering, Qingdao University of Science and Technology, Qingdao 266042, China

^b^ Institute of Chemical Industry of Forest Products, CAF, Nanjing 210042, China

**Supplementary Information**

1. The synthetic reaction path of [HO_3_S-(CH_2_)_3_-NEt_3_]Cl-ZnCl_2_

2. The IR spectral data of ILs

3. The ^1^HNMR of ILs

4. The IR of solid residues

1. The synthetic reaction path of [HO_3_S-(CH_2_)_3_-NEt_3_]Cl-ZnCl_2_

**Fig S1** ILs based on zinc chloride anions

2. The IR spectral data of ILs

[HO_3_S-(CH_2_)_3_-mim]Cl-FeCl_3_ (x=0.60): IR (KBr disc, cm^-1^) : v 3456, 3148, 3108, 2966, 2310, 1571, 1485, 1214, 1177, 1068, 850, 757, 695, 527.

[HO_3_S-(CH_2_)_3_-NEt_3_]Cl-FeCl_3_ (x=0.60): IR (KBr disc, cm^-1^) : v 3423, 2989, 1686, 1484, 1450, 1376, 1272, 1235,1158,1047,913,814,604,575, 501.

[HO_3_S-(CH_2_)_3_-py]Cl-FeCl_3_ (x=0.60): IR (KBr disc, cm^-1^) : v 3420, 3127, 2950, 1632, 1581, 1488, 1300, 1239, 1140, 1034, 830, 751, 608, 523.

3. The ^1^HNMR of ILs


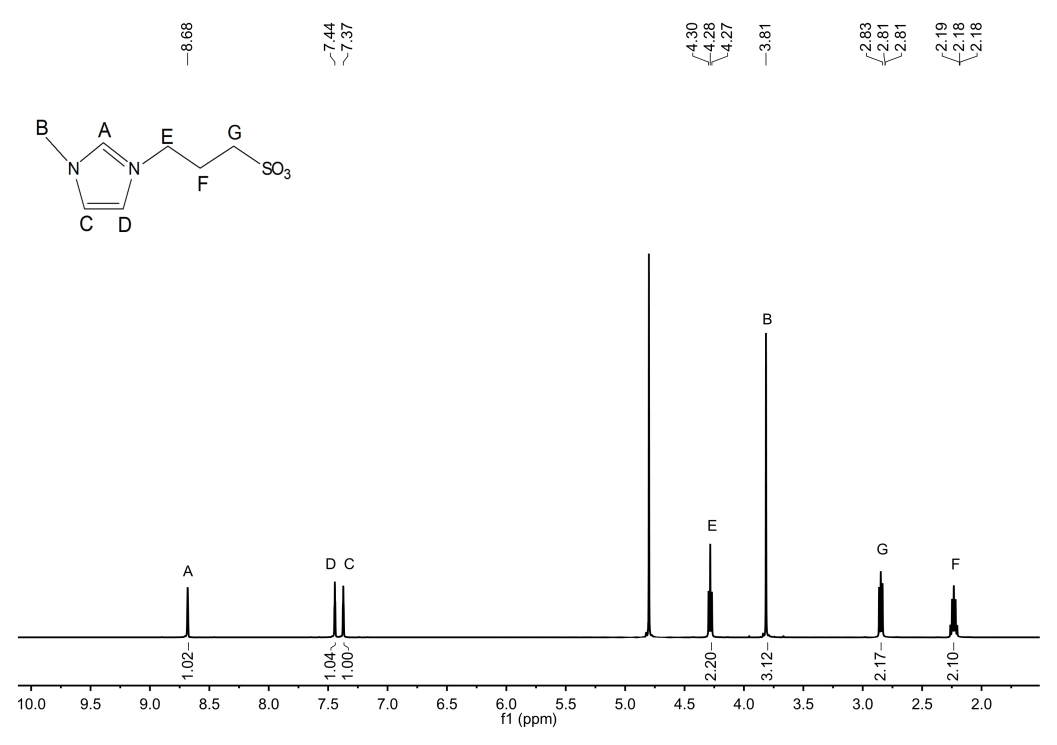


(a)


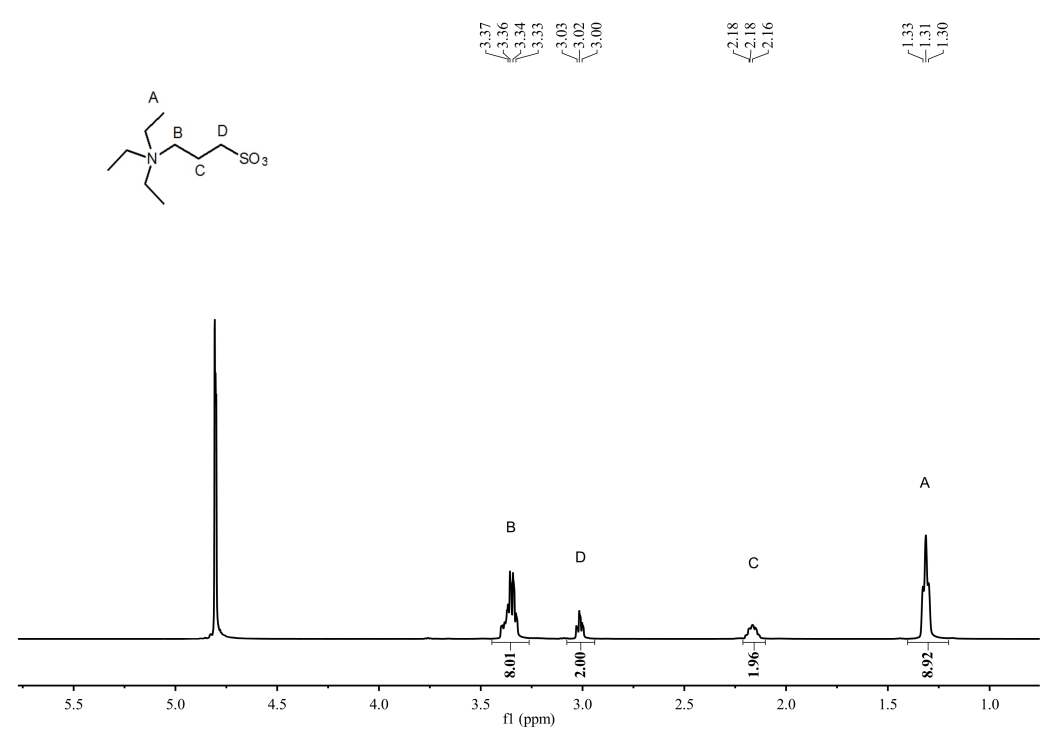


(b)


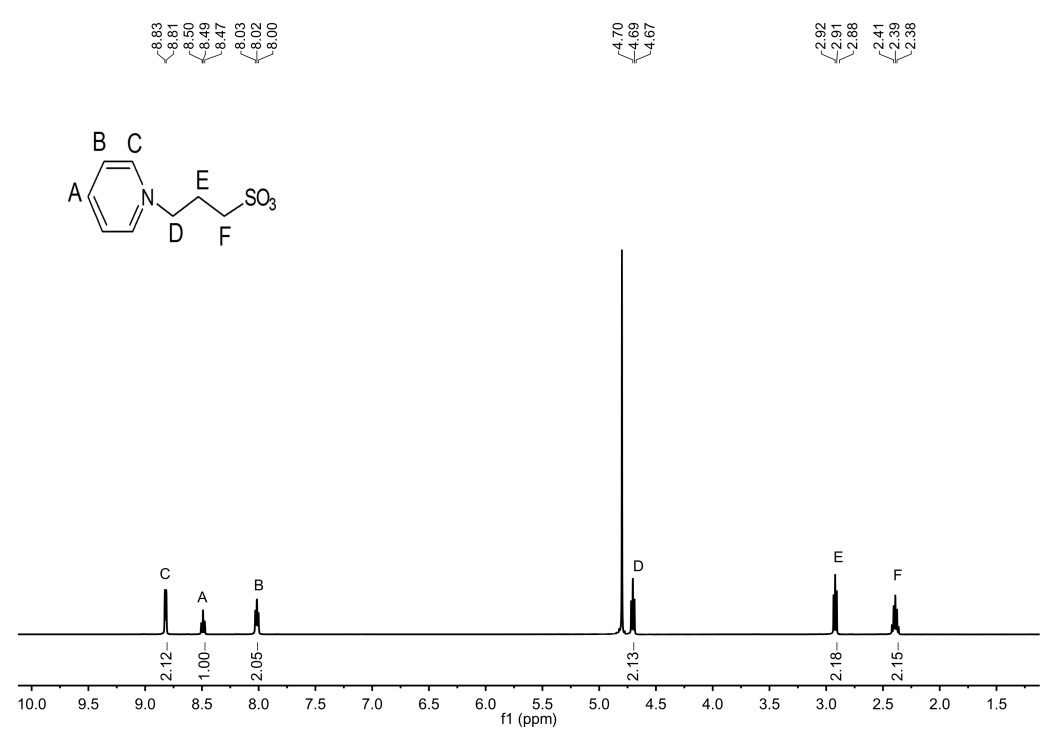


(c)

**Fig. S2** Typical ^1^H NMR spectra of ILs.

(a) [HO_3_S-(CH_2_)_3_-mim]Cl-FeCl_3_ (x=0.60)

(b) [HO_3_S-(CH_2_)_3_-NEt_3_]Cl-FeCl_3_ (x=0.60)

(c) [HO_3_S-(CH_2_)_3_-py]Cl-FeCl_3_ (x=0.60).

4. The IR of solid residues





**Fig. S3** IR spectrum of solid residues.

1. Corresponding author: 53 Zhengzhou Road, Qingdao, 266042, China; Fax: +86 532 84022719; [liushiweiqust@126.com](mailto:liushiweiqust@126.com). [↑](#footnote-ref-1)
2. Corresponding author: 53 Zhengzhou Road, Qingdao, 266042, China; Fax: +86 532 84022719;

   [yushitaoqust@126.com](mailto:yushitaoqust@126.com). [↑](#footnote-ref-2)
